# Risk of upper gastrointestinal bleeding in patients on oral anticoagulant and proton pump inhibitor co-therapy

**DOI:** 10.1371/journal.pone.0253310

**Published:** 2021-06-17

**Authors:** Hyun-Jung Lee, Hyung-Kwan Kim, Bong-Sung Kim, Kyung-Do Han, Jun-Bean Park, Heesun Lee, Seung-Pyo Lee, Yong-Jin Kim

**Affiliations:** 1 Division of Cardiology, Department of Internal Medicine, Cardiovascular Center, Seoul National University Hospital, Seoul, Korea; 2 Department of Statistics and Actuarial Science, Soongsil University, Seoul, Korea; East Tennessee State University, UNITED STATES

## Abstract

**Background:**

Proton pump inhibitors (PPIs) are known to reduce the risk of upper gastrointestinal bleeding in patients on oral anticoagulants, and patients are increasingly on oral anticoagulants and PPI co-therapy. However, evidence is lacking on the safety and effectiveness of oral anticoagulants when co-administered with PPIs.

**Methods:**

Among patients initiating oral anticoagulants (warfarin and non-vitamin K antagonist oral anticoagulants [NOACs], i.e. rivaroxaban, dabigatran, apixaban, and edoxaban) during 2013–2017, those concomitantly prescribed PPIs were identified (n = 19,851). The primary endpoint was hospitalization for major upper gastrointestinal bleeding, and secondary endpoints were death and ischemic stroke.

**Results:**

During a mean 1.4 years of follow-up, the primary endpoint occurred in 512 (2.58%) patients. Overall, NOACs were associated with lower upper gastrointestinal bleeding risk after adjustment for age, sex, comorbidities and concomitant medications (adjusted hazard ratio 0.78, 95% confidence interval 0.65–0.94), compared to warfarin. There was no significant difference in upper gastrointestinal bleeding risk among the individual NOACs. This trend of reduced risk for upper gastrointestinal bleeding in NOACs compared to warfarin was consistent for both regular and reduced doses, throughout bleeding risk groups, and other subgroup analyses. NOACs were also associated with lower risk of death compared to warfarin. The risk for ischemic stroke was not significantly different among the oral anticoagulants in patients with atrial fibrillation.

**Conclusion:**

In patients on oral anticoagulant and PPI co-therapy, NOACs were associated with lower risk of upper gastrointestinal bleeding and mortality compared to warfarin, while there was no difference among the oral anticoagulants for stroke prevention. In patients on PPI therapy, NOACs may preferred over warfarin for decreasing risk of upper gastrointestinal bleeding and mortality.

## Introduction

Age-related diseases including atrial fibrillation and cardiovascular diseases are on the rise worldwide with the progression of population aging [1, 2], and increasingly more people are on oral anticoagulants (OACs). Consequently, the bleeding risk associated with OACs is not negligible and attracts clinical attention. Proton pump inhibitors (PPIs) are recommended for OAC users on concomitant antiplatelet therapy to reduce upper gastrointestinal bleeding complications [3]. Previous observational studies and a randomized trial have also shown that PPIs are associated with reduced upper gastrointestinal bleeding risk in patients on OACs [4–6], suggesting that PPIs can be used to prevent upper gastrointestinal bleeding in OAC users. Meanwhile, there have also been concerns in pharmacodynamics and pharmacokinetic studies over possible drug interactions between PPIs and OACs, which are different for the individual OACs and are mostly related to dabigatran and warfarin [7–9]. While these interactions are presumed to have minor therapeutic effects [10], clinical outcomes data are hitherto lacking. Previous studies have reported differential risks in the gastrointestinal bleeding depending on the OACs [11–13]; however, no study has specifically focused on which OAC has the most favorable gastrointestinal safety profile in patients on OAC and PPI co-therapy.

We aimed to compare the upper gastrointestinal tract safety of OACs in a cohort of patients on OAC and PPI co-therapy. We also explored the effectiveness for stroke prevention and risk of death associated with OACs in these patients.

## Materials and methods

### Data source and study design

The study utilized the Korean National Health Insurance Service claims database covering the entire Korean population, the details of which have been described previously [14, 15]. This database is available for research on request at the National Health Insurance Sharing Service homepage (nhiss.nhis.or.kr), after study approval by the institutional review board approval and data provision review committee. We used a de-identified dataset conforming to the inclusion criteria detailed below (NHIS-2020-1-055), which is currently valid until August 19, 2021. The current study complied with the Declaration of Helsinki, and was approved by the Institutional Review Board of Seoul National University Hospital (E-1906-115-1041). Informed consent was waved due to the retrospective nature of the study and anonymized database.

**Fig 1:**
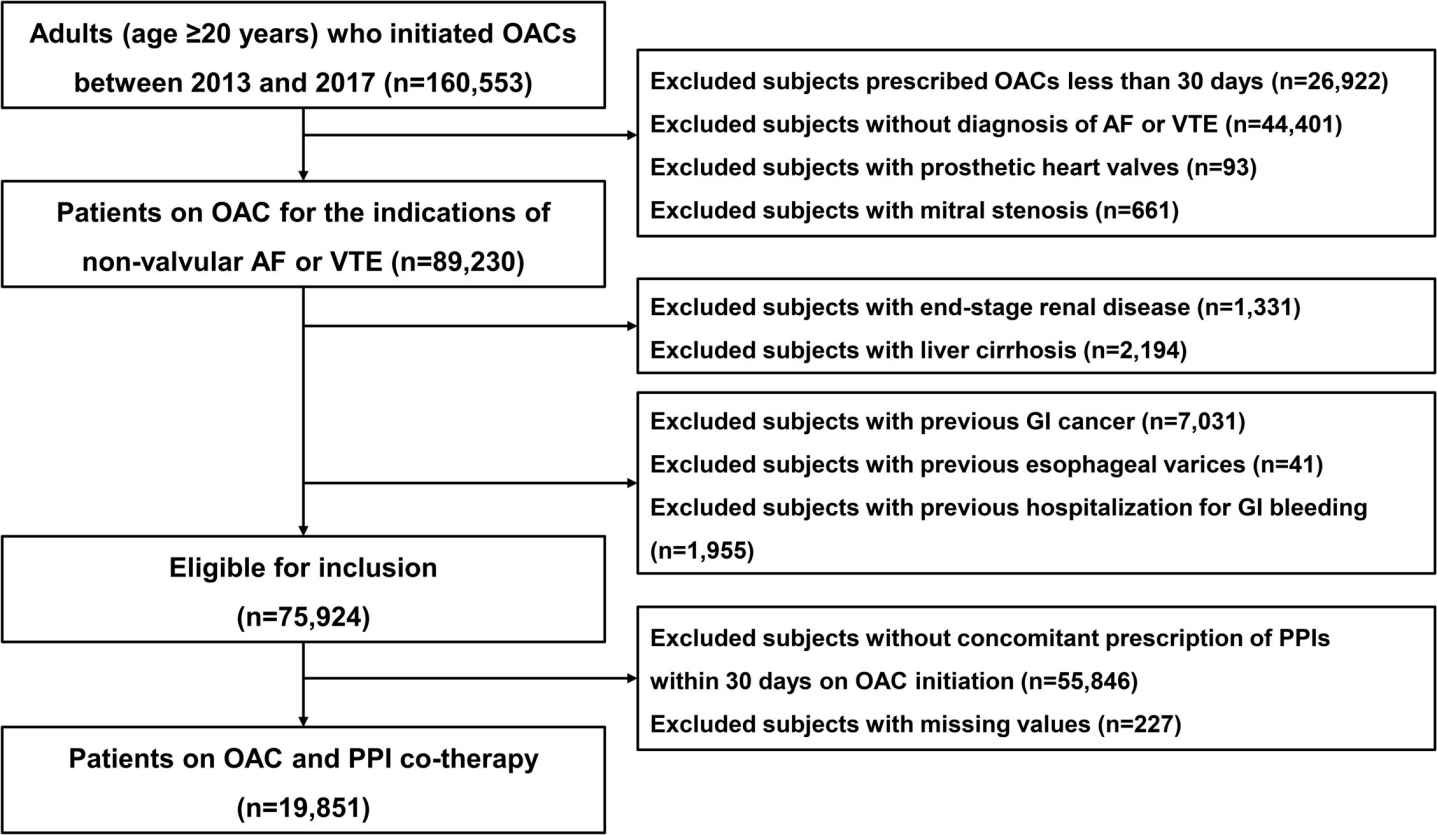
Study inclusion flow. OAC, oral anticoagulant; AF, atrial fibrillation; VTE, venous thromboembolism; GI, gastrointestinal; PPI, proton pump inhibitor.

We identified adults (age ≥20 years) who initiated OACs between January 2013 and December 2017, for the clinical indications of non-valvular atrial fibrillation or venous thromboembolism. According to regulations of the National Health Insurance Service, this population was provided after 50% random sampling from the database. OACs included warfarin and the non-vitamin K antagonist oral anticoagulants (NOACs), i.e. rivaroxaban, dabigatran, apixaban, and edoxaban. The starting year was set as 2013 which was when NOACs were approved for use in Korea. We excluded patients with previous prescription of OACs or less than 1 month of OAC use, and patients with prosthetic heart valves, mitral stenosis, end-stage renal disease or liver cirrhosis. We also excluded patients with very high gastrointestinal bleeding risk, i.e. those with previous gastrointestinal cancer, esophageal varices, or history of gastrointestinal bleeding within 1 year prior to OACs prescription. Finally, we included patients who were concomitantly prescribed PPIs within 1 month of OAC initiation (**Fig 1**). Proton pump inhibitors included pantoprazole, lansoprazole, omeprazole, rabeprazole, esomeprazole, ilaprazole, and dexlansoprazole.

### Covariates and study outcomes

Age, sex, and income level were ascertained from the database. Comorbidities were assessed for 1 year before the index OAC initiation. Diseases were defined using diagnostic codes, inpatient/outpatient hospital visits, and prescription codes (**S1 Table in S1 File**) [16, 17]. Concomitant antiplatelet and nonsteroidal anti-inflammatory drug (NSAID) use of at least 1 month or more during the study period was also identified. The HAS-BLED score was calculated as a measure of bleeding risk, by assessing the presence of hypertension (1 point), abnormal renal function (1 point; end-stage renal disease, chronic kidney disease, kidney transplantation), abnormal liver function (1 point; liver cirrhosis, liver disease), stroke (1 point; ischemic stroke), bleeding (1 point; previous hospitalization for gastrointestinal bleeding, peptic ulcer), old age (1 point; age >65 years), heavy alcohol drinking (1 point; >8 times/week), and antiplatelet or NSAID use (1 point) [18]. The CHA_2_DS_2_-VASc score was calculated as a measure of stroke risk in patients with atrial fibrillation, by assessing the presence of congestive heart failure (1 point), hypertension (1 point), old age (2 points if ≥75 years; 1 point if ≥65 years), diabetes mellitus (1 point), prior stroke or transient ischemic attack or systemic embolism (2 points), vascular disease (1 point; myocardial infarction or peripheral artery disease), and female sex (1 point) [17, 19] (**S1 Table in S1 File**).

The primary endpoint was major upper gastrointestinal bleeding, defined as hospitalization with red blood cell transfusion for bleeding related to gastric ulcers, duodenal ulcers, gastrojejunal ulcers, or hemorrhagic gastritis (**S1 Table in S1 File**). The secondary endpoint was death in the total study population, and the occurrence of ischemic stroke in the subpopulation of patients on OAC for non-valvular atrial fibrillation. The index date was the date of the OAC initiation and follow-up was until the occurrence of the endpoints, death, censoring due to discontinuation of OAC for more than 1 month or change of OAC type, or the end of the study (December 31, 2018).

### Statistical analysis

Categorical variables are presented as numbers (%), and continuous variables are presented as mean ± standard deviation or median (interquartile ranges). Incidence rates were calculated by dividing the number of events by the total follow-up period (per 1000 person-years). The risks of the upper gastrointestinal bleeding associated with warfarin, pooled NOACs, and individual NOACs were assessed with Cox regression analysis, and the hazard ratio (HR) and corresponding 95% confidence interval (CI) were estimated. Multivariable models were adjusted for age, sex, atrial fibrillation, venous thromboembolism, comorbidities including hypertension, diabetes mellitus, chronic kidney disease, ischemic heart disease, heart failure, and peptic ulcer disease, and the concomitant use of aspirin, P2Y_12_ inhibitor (clopidogrel, prasugrel, ticagrelor), and NSAID. Weighted cumulative incidence curves were drawn by the Kaplan-Meier method and compared with the log-rank test. We also performed separate analyses for the primary endpoint stratified by the NOAC dose regimen (i.e., regular and reduced). Pre-specified subgroup analyses were performed according to age, sex, indication for OAC use, number of antiplatelet agents, use of NSAID, history of peptic ulcer disease, and bleeding risk. We also conducted multivariable Cox regression analyses for the secondary endpoints of death and ischemic stroke.

We conducted sensitivity analyses to test the robustness of the main results. Due to the increasing popularity of NOACs, the number of patients prescribed warfarin decreased steadily while that of NOACs increased throughout the study period (**S2 Table in S1 File**). Thus, we performed a sensitivity analysis censoring patient follow-up at 2 years to approximate the follow-up durations of OAC groups (**S3 Table in S1 File**). Second, as the mortality rate was high within the study population, we conducted a sensitivity analysis considering death as a competing risk using the Fine-Gray method. Third, we conducted a sensitivity analysis in patients not on antiplatelets or NSAIDs.

A p-value <0.05 was considered statistically significant. SAS version 9.4 (SAS Institute, Cary, NC, USA) was used for all statistical analyses.

## Results

### Baseline characteristics

The study cohort included 19,851 patients on OAC and PPI co-therapy. There were 6,995 (35.2%), 6,468 (32.6%), 2,301 (11.6%), 2,613 (13.2%), and 1,474 (7.4%) patients initiating warfarin, rivaroxaban, dabigatran, apixaban, and edoxaban, respectively. Mean age was 71.0±12.7 years and 44.6% were men. Indication for OAC use was non-valvular atrial fibrillation in 63.5%, venous thromboembolism in 47.8%, and both in 11.3% of the patients. Approximately a third (31.2%) were on antiplatelet agents; 22.7% and 8.5% were on single and double antiplatelet therapy, respectively. NSAIDs were prescribed in 29.3% of the patients. Mean HAS-BLED score was 2.5±1.3, signifying that the study population had moderate bleeding risk.

Baseline characteristics according to the OAC groups are summarized in **Table 1**. Warfarin users were the youngest, while apixaban users were the oldest. Non-valvular atrial fibrillation was the main indication for OAC use except for rivaroxaban, the main indication of which was venous thromboembolism. Apixaban users had the highest proportion of comorbidities, with the only exception of previous history of peptic ulcer disease. Concomitant use of antiplatelet agents was highest in warfarin users (38.3%), while that of NSAIDs was highest in rivaroxaban users (33.6%). HAS-BLED scores were mostly similar among the OAC users, with apixaban users having a slightly higher score.

**Table 1:** Baseline characteristics of the study population.

	Warfarin (n = 6995)	Rivaroxaban (n = 6468)	Dabigatran (n = 2301)	Apixaban (n = 2613)	Edoxaban (n = 1474)	p-value
**Age (year)**						
Mean±SD	68.9±13.31	71.0±12.9	71.9±11.5	74.23±10.9	73.16±11.05	<0.001
≥75	2809 (40.2)	3005 (46.5)	1079 (46.9)	1496 (57.3)	760 (51.6)	<0.001
**Sex, male**	3367 (48.1)	2561 (39.6)	1118 (48.6)	1149 (44.0)	657 (44.6)	<0.001
**Income, low 20%**	1693 (24.2)	1611 (24.9)	548 (23.8)	573 (21.9)	357 (24.2)	0.056
**Comorbidities**						
Non-valvular atrial fibrillation	4681 (66.9)	2798 (43.3)	1790 (77.8)	2136 (81.8)	1200 (81.4)	<0.001
Venous thromboembolism	3125 (44.7)	4417 (68.3)	761 (33.1)	770 (29.5)	419 (28.4)	<0.001
Hypertension	4607 (65.9)	4181 (64.6)	1613 (70.1)	1910 (73.1)	1043 (70.8)	<0.001
Diabetes mellitus	1594 (22.8)	1416 (21.9)	544 (23.6)	681 (26.1)	341 (23.1)	<0.001
Chronic kidney disease	438 (6.2)	280 (4.3)	87 (3.8)	169 (6.5)	69 (4.7)	<0.001
Ischemic heart disease	2050 (29.3)	1675 (25.9)	695 (30.2)	883 (33.8)	473 (32.1)	<0.001
Heart failure	1748 (25.0)	1536 (23.8)	625 (27.2)	867 (33.2)	472 (32.0)	<0.001
Peptic ulcer disease	2109 (30.2)	1994 (30.8)	629 (27.3)	741 (28.4)	399 (27.1)	0.001
**Medications**						
Aspirin	2215 (31.7)	1396 (21.6)	446 (19.4)	475 (18.2)	250 (17.0)	<0.001
P2Y_12_ inhibitor	1347 (19.3)	829 (12.8)	320 (13.9)	383 (14.7)	207 (14.0)	<0.001
Number of antiplatelet agents						<0.001
0	4314 (61.7)	4627 (71.5)	1685 (73.2)	1932 (73.9)	1103 (74.8)	
1	1800 (25.7)	1457 (22.5)	466 (20.3)	504 (19.3)	285 (19.3)	
2	881 (12.6)	384 (5.9)	150 (6.5)	177 (6.8)	86 (5.8)	
NSAID	2026 (29.0)	2173 (33.6)	584 (25.4)	637 (24.4)	403 (27.3)	<0.001
Previous PPI use	3286 (47.0)	3705 (57.3)	1176 (51.1)	1408 (53.9)	861 (58.4)	<0.001
**HAS-BLED score**	2.50±1.31	2.51±1.29	2.49±1.22	2.63±1.22	2.53±1.21	0.001

NSAID, nonsteroidal anti-inflammatory drug; PPI, proton pump inhibitor.

### Risk of upper gastrointestinal bleeding

**Fig 2:**
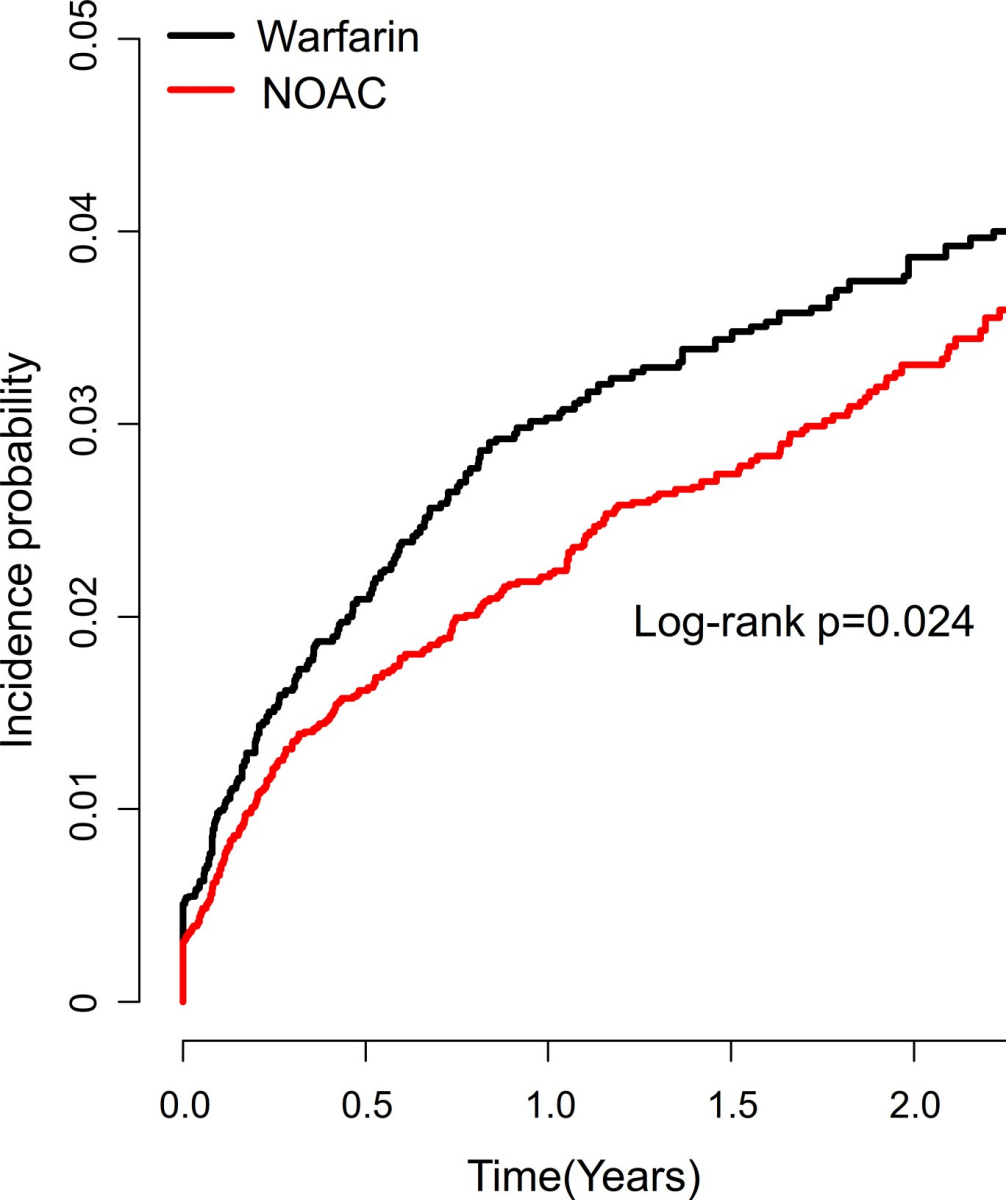
Weighted Kaplan-Meier curves for upper gastrointestinal bleeding events according to oral anticoagulants. NOAC, non-vitamin K antagonist oral anticoagulant.

**Fig 3:**
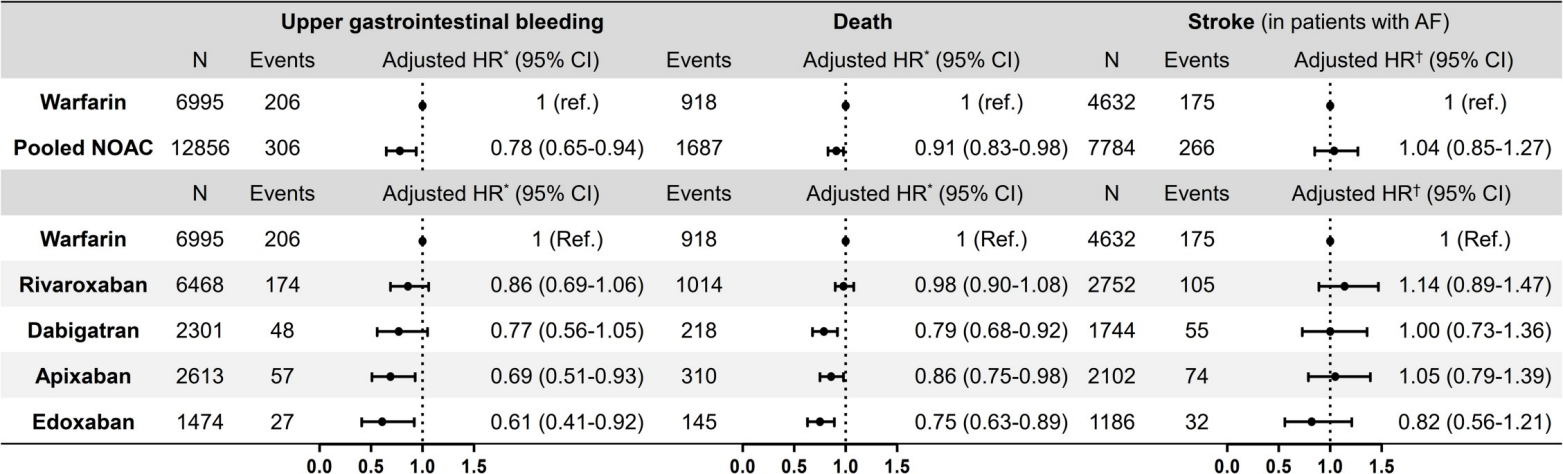
Differential risk of upper gastrointestinal bleeding, death, and ischemic stroke in users of warfarin and 4 NOACs. In patients on combined proton pump inhibitor and oral anticoagulant therapy, NOACs were associated with lower risk of upper gastrointestinal bleeding and mortality compared to warfarin, while there was no significant difference among the OACs in risk of stroke. *Adjusted for age, sex, atrial fibrillation, venous thromboembolism, comorbidities (hypertension, diabetes mellitus, chronic kidney disease, ischemic heart disease, heart failure, peptic ulcer disease), and concomitant use of aspirin, P2Y_12_ inhibitor, and nonsteroidal anti-inflammatory drug. ^†^Additionally adjusted for the CHA_2_DS_2_-VASc score. NOAC, non-vitamin K antagonist oral anticoagulant; AF, atrial fibrillation.

During a mean 1.41±1.35 years of follow-up, 512 (2.58%) patients on OAC and PPI co-therapy were hospitalized for major upper gastrointestinal bleeding. The weighted cumulative incidence of upper gastrointestinal bleeding events was higher in patients on warfarin compared to those on NOAC (**Fig 2**, log-rank p = 0.024). Weighted event-free survival for warfarin and NOAC users were 85.4% and 87% at 1 year, and 80.3% and 81.1% at 2 years, respectively. Compared to patients on warfarin, patients on NOACs had 22% lower risk of developing major upper gastrointestinal bleeding, after adjustment for age, sex, comorbidities and concomitant antiplatelets and NSAIDs (adjusted HR 0.78, 95% CI 0.65–0.94, p = 0.007) (**Fig 3, Table 2**). The largest risk reduction in upper gastrointestinal bleeding was observed in apixaban (adjusted HR 0.69, 95% CI 0.51–0.93, p = 0.014) and edoxaban users (adjusted HR 0.61, 95% CI 0.41–0.92, p = 0.019). There was no significant difference in upper gastrointestinal bleeding risk among the individual NOACs (p = 0.332), although adjusted HRs showed a progressively decreasing trend of events from rivaroxaban, dabigatran, apixaban, to edoxaban (**S4 Table in S1 File**).

**Table 2:** Upper gastrointestinal bleeding risk according to OAC treatment.

	N	Events	IR*	Crude HR (95% CI)	p-value		Adjusted HR^†^ (95% CI)	p-value	
Warfarin	6995	206	17.6	1 (reference)			1 (reference)		
NOACs	12856	306	18.8	0.91 (0.76–1.09)	0.324		0.78 (0.65–0.94)	0.007	
	N	Events	IR*	Crude HR (95% CI)	p-value	Overall p-value	Adjusted HR^†^ (95% CI)	p-value	Overall p-value
Warfarin	6995	206	17.6	1 (reference)		0.467	1 (reference)		0.032
Rivaroxaban	6468	174	19.1	0.98 (0.80–1.20)	0.858		0.86 (0.69–1.06)	0.145	
Dabigatran	2301	48	18.7	0.86 (0.63–1.18)	0.360		0.77 (0.56–1.05)	0.100	
Apixaban	2613	57	19.3	0.88 (0.65–1.18)	0.379		0.69 (0.51–0.93)	0.014	
Edoxaban	1474	27	16.6	0.72 (0.48–1.08)	0.109		0.61 (0.41–0.92)	0.019	

*IR: incidence rate per 1000 person-years.

^†^Adjusted for age, sex, atrial fibrillation, venous thromboembolism, hypertension, diabetes mellitus, chronic kidney disease, ischemic heart disease, heart failure, peptic ulcer disease, concomitant use of aspirin, P2Y_12_ inhibitor, and nonsteroidal anti-inflammatory drug.

Sensitivity analysis censoring follow-up at 2 years showed similar results (**S5, S6 Tables in S1 File**). In brief, patients on NOACs had lower upper gastrointestinal bleeding risk compared to those on warfarin (adjusted HR 0.78, 95% CI 0.64–0.94, p = 0.011). Sensitivity analysis accounting for the competing risk of death also showed consistent results (adjusted HR 0.77, 95% CI 0.64–0.92, p = 0.004; **S7, S8 Tables in S1 File**). Sensitivity analysis excluding patients on antiplatelet agents or NSAIDs also showed that the use of NOAC was associated with lower upper gastrointestinal bleeding risk compared to use of warfarin (adjusted HR 0.74, 95% CI 0.58–0.95, p = 0.020; **S9, S10 Tables in S1 File**). In all the sensitivity analyses as well, upper gastrointestinal bleeding risks among the individual NOACs were not significantly different (p = 0.216, p = 0.334, and p = 0.268, respectively), though there was a trend for decreasing adjusted HRs in the order of rivaroxaban, dabigatran, apixaban, and edoxaban (**S6, S8, and S10 Tables in S1 File**).

**Fig 4:**
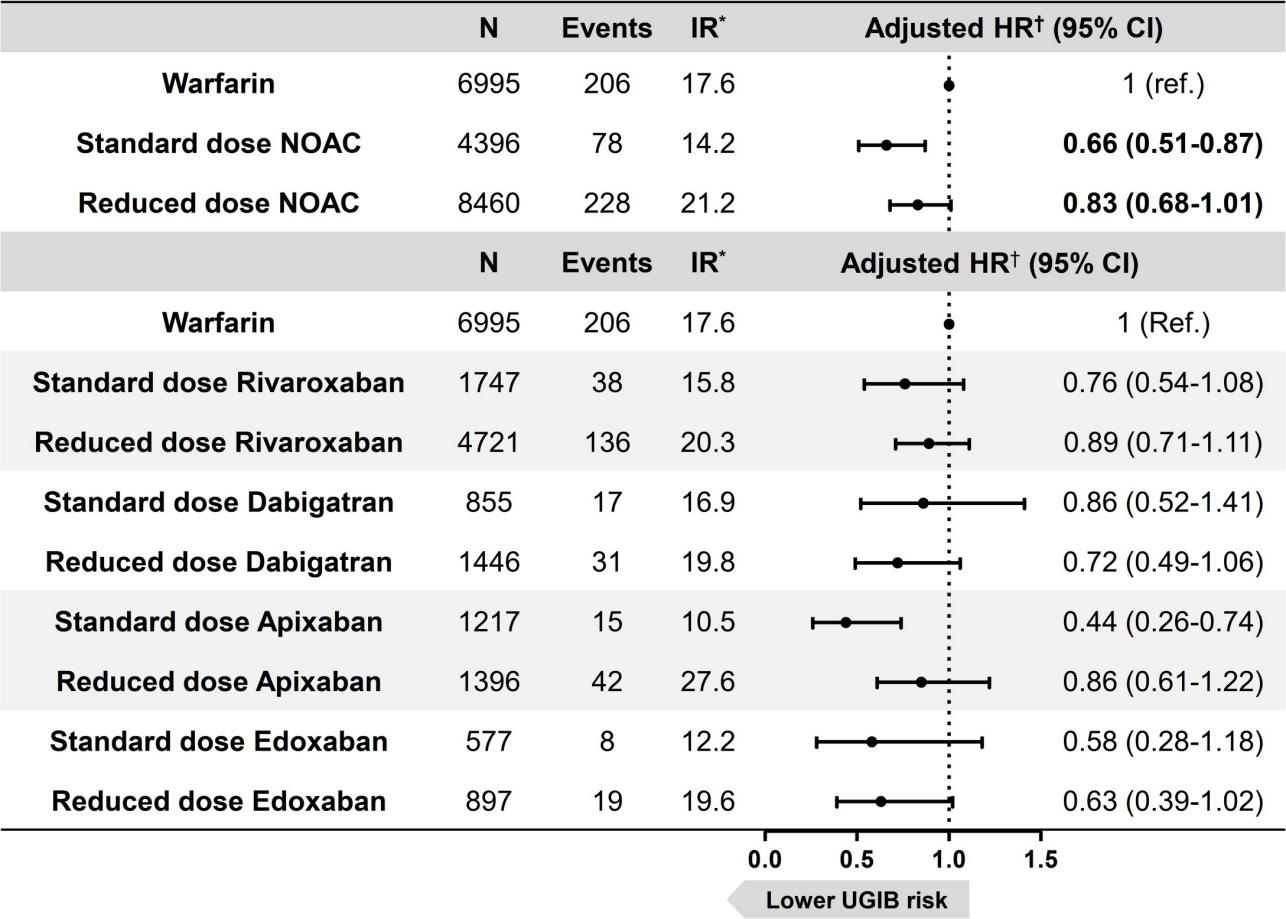
Comparison of upper gastrointestinal bleeding risk in users of warfarin and 4 NOACs with consideration of dose regimens. *IR: incidence rate per 1000 person-years. ^†^Adjusted for age, sex, atrial fibrillation, venous thromboembolism, comorbidities (hypertension, diabetes mellitus, chronic kidney disease, ischemic heart disease, heart failure, peptic ulcer disease), and concomitant use of aspirin, P2Y_12_ inhibitor, and nonsteroidal anti-inflammatory drug. NOAC, non-vitamin K antagonist oral anticoagulant; UGIB, upper gastrointestinal bleeding.

Among NOAC users, 65.8% (n = 8,460) were prescribed reduced dose of NOACs. Reduced doses of NOACs were defined as 15 or 10 mg rivaroxaban once daily, 110 mg dabigatran twice daily, 2.5 mg apixaban twice daily, and 30 mg edoxaban once daily, while regular doses of NOACs were defined as 20 mg rivaroxaban once daily, 150 mg dabigatran twice daily, 5 mg apixaban twice daily, and 60 mg edoxaban once daily. Compared to patients on warfarin, there was a tendency for reduced upper gastrointestinal bleeding risk in patients on both standard dose and reduced dose of NOACs, which was statistically significant in standard dose of NOACs and was of borderline significance in reduced dose of NOACs (**Fig 4**). This trend of reduced upper gastrointestinal bleeding risk compared to warfarin was consistent for both regular and reduced dose regimens of all individual NOACs.

**Fig 5:**
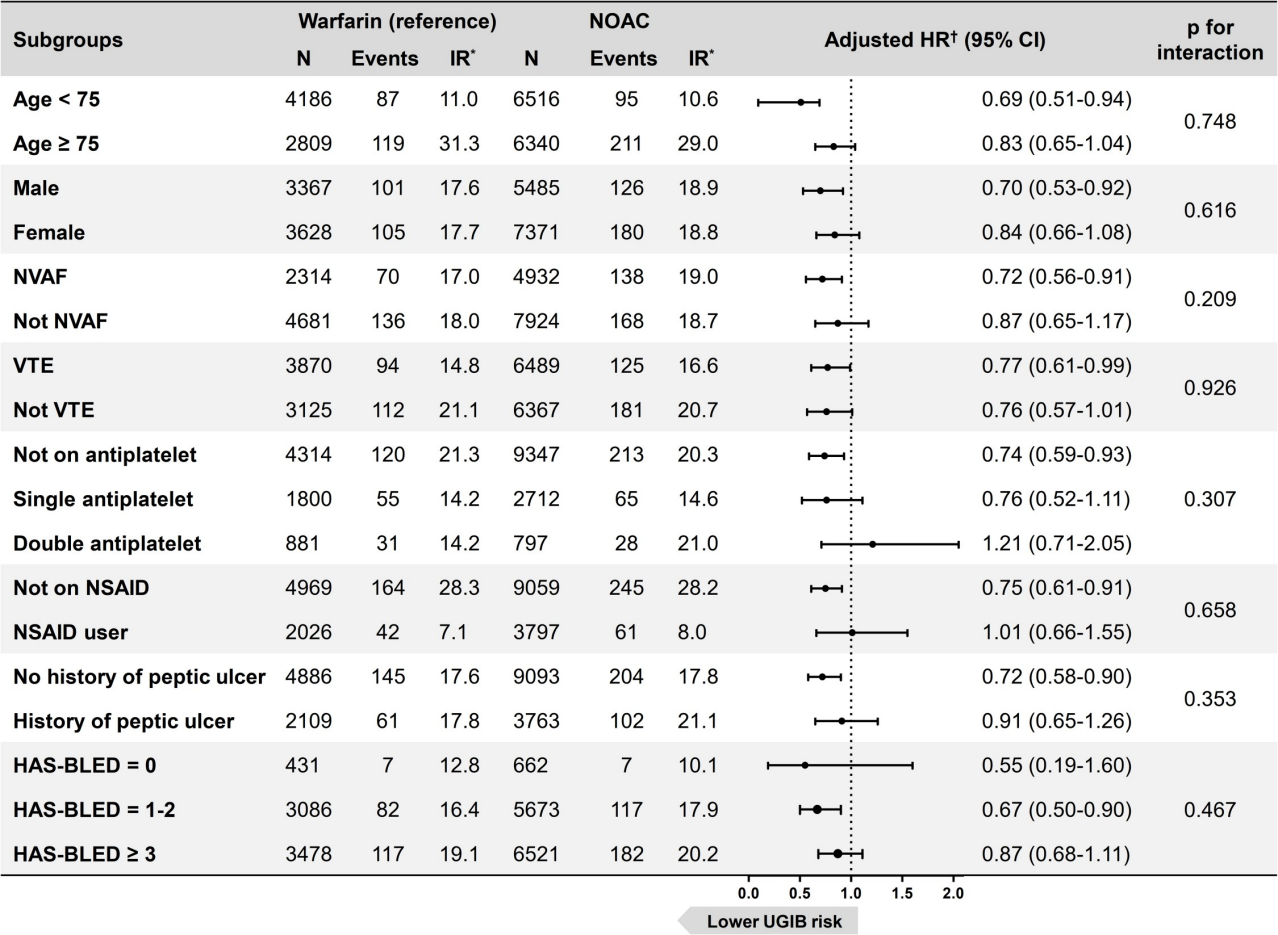
Upper gastrointestinal bleeding risk of NOACs with reference to warfarin in various subgroups. *IR: incidence rate per 1000 person-years. ^†^Adjusted for age, sex, atrial fibrillation, venous thromboembolism, comorbidities (hypertension, diabetes mellitus, chronic kidney disease, ischemic heart disease, heart failure, peptic ulcer disease), and concomitant use of aspirin, P2Y_12_ inhibitor, and nonsteroidal anti-inflammatory drug. NOAC, non-vitamin K antagonist oral anticoagulant; NSAID, nonsteroidal anti-inflammatory drug; NVAF, non-valvular atrial fibrillation; UGIB, upper gastrointestinal bleeding; VTE, venous thromboembolism.

In subgroup analyses according to age (<75, ≥75), sex, indication for OAC use (i.e. non-valvular atrial fibrillation or venous thromboembolism), number of antiplatelet agents (0, 1, or 2), NSAID use, history of peptic ulcer disease, and bleeding risk according to the HAS-BLED scores (0, 1–2, ≥3), tests for interaction showed no significant subgroup differences in the treatment effects of OACs (**Fig 5**). There was a general trend for reduced upper gastrointestinal bleeding risk in NOAC users compared to warfarin users, which was consistent throughout the subgroups, except for similar risks in patients on 2 antiplatelet agents or NSAIDs. Notably, the trend of lower gastrointestinal bleeding risk associated with NOACs compared to warfarin was consistent throughout patients with low (0), moderate (1–2), or high bleeding risk (≥3) by HAS-BLED scores; this trend was also consistent for individual NOACs (**S1 Fig in S1 File**).

### Risk of death

During a mean 1.43±1.35 years of follow-up, 2605 (13.1%) deaths occurred in patients on OAC and PPI co-therapy. Compared to patients on warfarin, patients on NOACs had a small but significant reduction in the risk of death, after adjustment for age, sex, comorbidities and concomitant medication (adjusted HR 0.91, 95% CI 0.83–0.98, p = 0.020) (**Table 3**). The lower risk of death was significant in dabigatran (adjusted HR 0.79, 95% CI 0.68–0.92, p = 0.002), apixaban (adjusted HR 0.86, 95% CI 0.75–0.98, p = 0.023), and edoxaban (adjusted HR 0.75, 95% CI 0.63–0.89, p = 0.001) users. Among individual NOACs, patients on dabigatran (adjusted HR 0.83, 95% CI 0.71–0. 97, p = 0.016) and edoxaban (adjusted HR 0.81, 95% CI 0.67–0.96, p = 0.019) had lower risk of death compared to patients on rivaroxaban (**S11 Table in S1 File**).

**Table 3:** Risk of death according to OAC treatment.

	N	Events	IR*	Crude HR (95% CI)	p-value		Adjusted HR^†^ (95% CI)	p-value	
Warfarin	6995	918	77.3	1 (reference)			1 (reference)		
NOACs	12856	1687	102.4	1.19 (1.10–1.29)	<0.001		0.91 (0.83–0.98)	0.020	
	N	Events	IR*	Crude HR (95% CI)	p-value	Overall p-value	Adjusted HR^†^ (95% CI)	p-value	Overall p-value
Warfarin	6995	918	77.3	1 (reference)		<0.001	1 (reference)		<0.001
Rivaroxaban	6468	1014	109.4	1.32 (1.21–1.45)	<0.001		0.98 (0.90–1.08)	0.720	
Dabigatran	2301	218	83.9	0.95 (0.82–1.10)	0.471		0.79 (0.68–0.92)	0.002	
Apixaban	2613	310	104.3	1.15 (1.01–1.31)	0.040		0.86 (0.75–0.98)	0.023	
Edoxaban	1474	145	88.4	0.93 (0.78–1.10)	0.390		0.75 (0.63–0.89)	0.001	

Footnotes same as Table 2.

### Risk of stroke

To compare the effectiveness of OACs when administered concomitantly with PPIs, the risk of ischemic stroke was compared in the subpopulation of patients on OAC for the indication of non-valvular atrial fibrillation (n = 12,416). Baseline characteristics according to the OAC used in this subpopulation are summarized in **S12 Table in S1 File**, and were similar to those of the total study population. The mean CHA_2_DS_2_-VASc score was 3.6±2.1, and was highest in apixaban users (3.9±2.0) and lowest in warfarin users (3.3±2.1). Ischemic stroke occurred in 441 (3.6%) patients. There was no significant difference in the risk of ischemic stroke between patients on warfarin and those on NOAC, after adjustment for age, sex, comorbidities, concomitant medication, and the CHA_2_DS_2_-VASc score (p = 0.719; **Table 4**). There was no significant difference among the individual NOACs, as well (p = 0.374; **S13 Table in S1 File**).

**Table 4:** Risk of stroke according to OAC treatment in patients with atrial fibrillation.

	N	Events	IR*	Crude HR (95% CI)	p-value		Adjusted HR^†^ (95% CI)	p-value	
Warfarin	4632	175	23.3	1 (reference)			1 (reference)		
NOAC	7784	266	29.7	1.07 (0.88–1.30)	0.494		1.04 (0.85–1.27)	0.719	
	N	Events	IR*	Crude HR (95% CI)	p-value	Overall p-value	Adjusted HR^†^ (95% CI)	p-value	Overall p-value
Warfarin	4632	175	23.3	1 (reference)		0.458	1 (reference)		0.560
Rivaroxaban	2752	105	31.8	1.17 (0.92–1.50)	0.203		1.14 (0.89–1.47)	0.295	
Dabigatran	1744	55	28.4	1.01 (0.75–1.38)	0.932		1.00 (0.73–1.36)	0.983	
Apixaban	2102	74	31.3	1.11 (0.84–1.46)	0.455		1.05 (0.79–1.39)	0.769	
Edoxaban	1186	32	23.8	0.84 (0.57–1.22)	0.358		0.82 (0.56–1.21)	0.315	

*IR: incidence rate per 1000 person-years.

^†^Adjusted for age, sex, hypertension, diabetes mellitus, chronic kidney disease, ischemic heart disease, heart failure, peptic ulcer disease, concomitant use of aspirin, P2Y_12_ inhibitor, nonsteroidal anti-inflammatory drug, and CHA_2_DS_2_-VASc score.

## Discussion

In a large cohort of patients on OAC and PPI co-therapy, significant differences were clinically observed in upper gastrointestinal bleeding risk according to the OACs used. Specifically, NOACs were associated with lower risk of upper gastrointestinal bleeding compared to warfarin, after multivariable adjustment for age, sex, baseline comorbidities, and concomitant medication including antiplatelet agents and NSAIDs. The largest reduction in upper gastrointestinal bleeding risk was noted with apixaban and edoxaban, as compared to warfarin. Among four individual NOACs, the difference in upper gastrointestinal bleeding risk was not statistically significant. The trend of reduced risk for upper gastrointestinal bleeding in NOACs compared to warfarin was consistent for both regular and reduced dose NOAC regimens, and in subgroup analyses stratified by age, sex, indication for OAC use, number of antiplatelet agents or NSAID use, and history of peptic ulcer disease. In addition, NOACs were associated with lower risk of death compared to warfarin. Meanwhile, there was no significant difference in risk of ischemic stroke among the OACs in patients with atrial fibrillation. To our knowledge, this is the first study comparing the clinical outcomes of patients on OAC and PPI co-therapy.

PPIs have been associated with reduced risk of upper gastrointestinal bleeding in patients on OACs, especially in high-risk patients [4–6]. Guidelines also recommend co-administration of PPIs in OAC users on concomitant antiplatelet therapy [3]. Age-related cardiovascular diseases and atrial fibrillation are on the rise in the aging society [1, 2], while aging itself also predisposes to higher risk of gastrointestinal bleeding complications associated with antithrombotic therapy [20]. Thus, we can expect that PPIs will be prescribed more frequently to prevent upper gastrointestinal bleeding in OAC users, especially in those with higher bleeding risk. However, there is a lack of data on the optimal OAC choice considering the gastrointestinal safety and effectiveness of individual OACs in patients on OAC and PPI co-therapy. Although previous studies have shown that there are differences in gastrointestinal bleeding risk among the OACs without considering PPI use [11–13], it is unclear whether we can extrapolate these clinical observations that compared gastrointestinal safety in general OAC users to the subpopulation of patients on OAC and PPI co-therapy.

Some evidence suggests that the gastrointestinal safety of OACs may be altered with the concomitant use of PPIs. According to a previous study showing that PPI use was associated with reduced upper gastrointestinal bleeding events for all OACs, this risk reduction was most pronounced in dabigatran [5]. Dabigatran has a tartaric acid core that may cause dyspepsia and characteristic upper gastrointestinal tract mucosal injury [21, 22], and theoretically PPIs could prevent upper gastrointestinal bleeding events related to these lesions. Meanwhile, other NOACs such as rivaroxaban, apixaban, and edoxaban do not have a tartaric acid core and have not been linked to such mucosal erosions, except for one case report with rivaroxaban [23]. Also, co-administration of PPIs was reported to decrease the bioavailability of dabigatran around 12~24% [9, 10, 24, 25], which may be related to lower gastrointestinal bleeding risk but also potentially reduced effectiveness for preventing stroke or venous thromboembolism. The absorption of dabigatran is enhanced by a low pH [26], and thus can be hindered by PPI co-administration. On the other hand, the pharmacokinetics of rivaroxaban, apixaban, and edoxaban are not affected by gastric pH modifiers [8, 27, 28]. PPIs have also been suggested to potentiate the anticoagulant effects of warfarin, and caution should be taken to carefully monitor patients on warfarin and PPI co-therapy [7]. This may be related to the increased absorption of warfarin in a higher pH, and reduced metabolism of warfarin due to altered cytochrome P activity by the PPIs [29, 30]. However, drug interactions may differ according to PPI type [30], and some studies suggest that there are no clinically meaningful interactions between PPIs and warfarin [31, 32]. Fortunately, laboratory monitoring with prothrombin time makes it possible to adjust the level of anticoagulation in patients on warfarin, which is not possible in those on NOACs. Meanwhile, there is a lack of data on whether expected drug interactions between PPIs and OACs translate into clinically significant differences in patient outcome.

Previous studies have shown differences in the gastrointestinal bleeding risk of OACs [11–13]. In a meta-analysis using the landmark NOAC clinical trials, NOACs reduced ischemic stroke or systemic embolism, but were associated with increased gastrointestinal bleeding compared to warfarin [11]. In another meta-analysis including 43 randomized clinical trials, pooled NOACs and warfarin showed no differences in upper gastrointestinal bleeding, and rivaroxaban and dabigatran were associated with increased risk of major gastrointestinal bleeding, which was not true for apixaban and edoxaban [12]. Meanwhile, another meta-analysis of the landmark trials suggested that NOACs increased gastrointestinal bleeding compared to warfarin in non-Asians, but not in Asians [33]. In observational studies comparing individual NOACs, apixaban had the lowest gastrointestinal bleeding risk, and was significantly lower than that of rivaroxaban or dabigatran [13, 34, 35]. In Asians, NOACs were associated with lower risks of gastrointestinal bleeding and ischemic stroke compared to warfarin, with apixaban showing the lowest rates of gastrointestinal bleeding [13]. Our study showed that in patients on OAC and PPI co-therapy, warfarin was related to a higher upper gastrointestinal bleeding risk compared to those on NOACs. Although the difference among the individual NOACs were not statistically significant, there was a trend for the lowest gastrointestinal bleeding risk in apixaban and edoxaban. Moreover, NOACs were associated with reduced mortality compared to warfarin. In contrary to the previous studies that showed lower risk of stroke with NOACs compared to warfarin [11, 13, 33], we found no significant difference in risk for ischemic stroke between the NOACs and warfarin when PPI was co-administrated. In the case of dabigatran, this may be related to lower bioavailability due to PPI co-therapy, while the same can be prevented by prothrombin time monitoring in warfarin, as described above. There is a possibility that the co-administration of PPI may reduce the effectiveness of other NOACs as well, through yet unknown mechanisms. To note, another study suggested that the effectiveness of NOACs for stroke prevention may be decreased compared with warfarin in patients with increasing polypharmacy, especially that of apixaban [36].

This study has several limitations that need to be addressed. First, possible differences with individual PPIs could not be analyzed due to the reduction in statistical power. Second, we excluded patients with gastrointestinal cancer, esophageal varices, or recent history of gastrointestinal bleeding, and this may limit generalization of the study results to these patients at very high risk for bleeding. Third, the time in therapeutic range in warfarin users could not be evaluated, as prothrombin time was unavailable in this database. Fourth, information on concomitant steroid use was unavailable. Also, possible drug interaction with other drugs were not considered in analysis. Fifth, while we performed multivariable adjustment for differences in observed covariates, there may be residual confounding related to unmeasured covariates. Finally, this is a retrospective study with inherent limitations and while demonstrating association, it does not prove causality.

In conclusion, in patients on OAC and PPI co-therapy, NOACs were associated with reduced upper gastrointestinal bleeding compared to warfarin, especially apixaban and edoxaban. NOACs were also associated with lower mortality compared to warfarin. There was no significant difference in risk for ischemic stroke among the OACs in patients with non-valvular atrial fibrillation. Results from observational studies suggest that PPIs can reduce gastrointestinal bleeding in patients on oral anticoagulation, especially in high-risk patients. If these results are confirmed in randomized controlled trials, the combined use of OAC and PPI may become a part of routine clinical practice. However, it is yet unclear which OAC regimen is optimal in patients on PPI therapy. The results of this study suggest that NOACs may be preferred to warfarin for better safety issues without difference in effectiveness. Further prospective studies are needed to confirm these results.

## Supporting information

S1 File Supplemental materials. S1 Fig, S1-S13 Tables.
(PDF)

## References

[1] Paneni F, Diaz Canestro C, Libby P, Luscher TF, Camici GG. The Aging Cardiovascular System: Understanding It at the Cellular and Clinical Levels. J Am Coll Cardiol. 2017;69(15):1952–67. doi: 10.1016/j.jacc.2017.01.064 28408026

[2] Aronow WS, Banach M. Atrial Fibrillation: The New Epidemic of the Ageing World. J Atr Fibrillation. 2009;1(6):154. doi: 10.4022/jafib.154 28496617 PMC5398780

[3] Bhatt DL, Scheiman J, Abraham NS, Antman EM, Chan FK, Furberg CD, et al. ACCF/ACG/AHA 2008 expert consensus document on reducing the gastrointestinal risks of antiplatelet therapy and NSAID use: a report of the American College of Cardiology Foundation Task Force on Clinical Expert Consensus Documents. J Am Coll Cardiol. 2008;52(18):1502–17. doi: 10.1016/j.jacc.2008.08.002 19017521

[4] Chan EW, Lau WC, Leung WK, Mok MT, He Y, Tong TS, et al. Prevention of Dabigatran-Related Gastrointestinal Bleeding With Gastroprotective Agents: A Population-Based Study. Gastroenterology. 2015;149(3):586–95 e3. doi: 10.1053/j.gastro.2015.05.002 25960019

[5] Ray WA, Chung CP, Murray KT, Smalley WE, Daugherty JR, Dupont WD, et al. Association of Oral Anticoagulants and Proton Pump Inhibitor Cotherapy With Hospitalization for Upper Gastrointestinal Tract Bleeding. JAMA. 2018;320(21):2221–30. doi: 10.1001/jama.2018.17242 30512099 PMC6404233

[6] Moayyedi P, Eikelboom JW, Bosch J, Connolly SJ, Dyal L, Shestakovska O, et al. Pantoprazole to Prevent Gastroduodenal Events in Patients Receiving Rivaroxaban and/or Aspirin in a Randomized, Double-Blind, Placebo-Controlled Trial. Gastroenterology. 2019;157(2):403–12 e5. doi: 10.1053/j.gastro.2019.04.041 31054846

[7] Agewall S, Cattaneo M, Collet JP, Andreotti F, Lip GY, Verheugt FW, et al. Expert position paper on the use of proton pump inhibitors in patients with cardiovascular disease and antithrombotic therapy. Eur Heart J. 2013;34(23):1708–13, 13a-13b. doi: 10.1093/eurheartj/eht042 23425521

[8] Gelosa P, Castiglioni L, Tenconi M, Baldessin L, Racagni G, Corsini A, et al. Pharmacokinetic drug interactions of the non-vitamin K antagonist oral anticoagulants (NOACs). Pharmacol Res. 2018;135:60–79. doi: 10.1016/j.phrs.2018.07.016 30040996

[9] Kuwayama T, Osanai H, Ajioka M, Tokuda K, Ohashi H, Tobe A, et al. Influence of proton pump inhibitors on blood dabigatran concentrations in Japanese patients with non-valvular atrial fibrillation. J Arrhythm. 2017;33(6):619–23. doi: 10.1016/j.joa.2017.07.013 29255511 PMC5729000

[10] Liesenfeld KH, Lehr T, Dansirikul C, Reilly PA, Connolly SJ, Ezekowitz MD, et al. Population pharmacokinetic analysis of the oral thrombin inhibitor dabigatran etexilate in patients with non-valvular atrial fibrillation from the RE-LY trial. J Thromb Haemost. 2011;9(11):2168–75. doi: 10.1111/j.1538-7836.2011.04498.x 21972820

[11] Ruff CT, Giugliano RP, Braunwald E, Hoffman EB, Deenadayalu N, Ezekowitz MD, et al. Comparison of the efficacy and safety of new oral anticoagulants with warfarin in patients with atrial fibrillation: a meta-analysis of randomised trials. Lancet. 2014;383(9921):955–62. doi: 10.1016/S0140-6736(13)62343-0 24315724

[12] Miller CS, Dorreen A, Martel M, Huynh T, Barkun AN. Risk of Gastrointestinal Bleeding in Patients Taking Non-Vitamin K Antagonist Oral Anticoagulants: A Systematic Review and Meta-analysis. Clin Gastroenterol Hepatol. 2017;15(11):1674–83 e3. doi: 10.1016/j.cgh.2017.04.031 28458008

[13] Lee SR, Choi EK, Kwon S, Han KD, Jung JH, Cha MJ, et al. Effectiveness and Safety of Contemporary Oral Anticoagulants Among Asians With Nonvalvular Atrial Fibrillation. Stroke. 2019;50(8):2245–9. doi: 10.1161/STROKEAHA.119.025536 31208303

[14] Lee HJ, Lee SR, Choi EK, Han KD, Oh S. Low Lipid Levels and High Variability are Associated With the Risk of New-Onset Atrial Fibrillation. J Am Heart Assoc. 2019;8(23):e012771. doi: 10.1161/JAHA.119.012771 31771440 PMC6912974

[15] Song SO, Jung CH, Song YD, Park CY, Kwon HS, Cha BS, et al. Background and data configuration process of a nationwide population-based study using the korean national health insurance system. Diabetes Metab J. 2014;38(5):395–403. doi: 10.4093/dmj.2014.38.5.395 25349827 PMC4209354

[16] Lee HJ, Kim HK, Jung JH, Han KD, Lee H, Park JB, et al. Novel Oral Anticoagulants for Primary Stroke Prevention in Hypertrophic Cardiomyopathy Patients With Atrial Fibrillation. Stroke. 2019;50(9):2582–6. doi: 10.1161/STROKEAHA.119.026048 31340730

[17] Lee SR, Lee HJ, Choi EK, Han KD, Jung JH, Cha MJ, et al. Direct Oral Anticoagulants in Patients With Atrial Fibrillation and Liver Disease. J Am Coll Cardiol. 2019;73(25):3295–308. doi: 10.1016/j.jacc.2019.04.052 31248551

[18] Pisters R, Lane DA, Nieuwlaat R, de Vos CB, Crijns HJ, Lip GY. A novel user-friendly score (HAS-BLED) to assess 1-year risk of major bleeding in patients with atrial fibrillation: the Euro Heart Survey. Chest. 2010;138(5):1093–100. doi: 10.1378/chest.10-0134 20299623

[19] Lip GY, Nieuwlaat R, Pisters R, Lane DA, Crijns HJ. Refining clinical risk stratification for predicting stroke and thromboembolism in atrial fibrillation using a novel risk factor-based approach: the euro heart survey on atrial fibrillation. Chest. 2010;137(2):263–72. doi: 10.1378/chest.09-1584 19762550

[20] Greenwald DA. Aging, the gastrointestinal tract, and risk of acid-related disease. Am J Med. 2004;117 Suppl 5A:8S–13S. doi: 10.1016/j.amjmed.2004.07.019 15478847

[21] Toya Y, Nakamura S, Tomita K, Matsuda N, Abe K, Abiko Y, et al. Dabigatran-induced esophagitis: The prevalence and endoscopic characteristics. J Gastroenterol Hepatol. 2016;31(3):610–4. doi: 10.1111/jgh.13024 26102078

[22] Matsuura H, Suto K, Yasuhara H, Hata H. Longitudinal sloughing mucosal casts: dabigatran-induced oesophagitis. Eur Heart J. 2018;39(36):3400. doi: 10.1093/eurheartj/ehy538 30169635

[23] Cox R, Roche E, Fairley S. Novel oral anticoagulant drugs and severe oesophagitis dissecans. Intern Med J. 2016;46(12):1456–7. doi: 10.1111/imj.13275 27981772

[24] Stangier J, Eriksson BI, Dahl OE, Ahnfelt L, Nehmiz G, Stahle H, et al. Pharmacokinetic profile of the oral direct thrombin inhibitor dabigatran etexilate in healthy volunteers and patients undergoing total hip replacement. J Clin Pharmacol. 2005;45(5):555–63. doi: 10.1177/0091270005274550 15831779

[25] Stangier J, Stahle H, Rathgen K, Fuhr R. Pharmacokinetics and pharmacodynamics of the direct oral thrombin inhibitor dabigatran in healthy elderly subjects. Clin Pharmacokinet. 2008;47(1):47–59. doi: 10.2165/00003088-200847010-00005 18076218

[26] Connolly SJ, Ezekowitz MD, Yusuf S, Eikelboom J, Oldgren J, Parekh A, et al. Dabigatran versus warfarin in patients with atrial fibrillation. N Engl J Med. 2009;361(12):1139–51. doi: 10.1056/NEJMoa0905561 19717844

[27] Moore KT, Plotnikov AN, Thyssen A, Vaccaro N, Ariyawansa J, Burton PB. Effect of multiple doses of omeprazole on the pharmacokinetics, pharmacodynamics, and safety of a single dose of rivaroxaban. J Cardiovasc Pharmacol. 2011;58(6):581–8. doi: 10.1097/FJC.0b013e31822f6c2b 21822144

[28] Upreti VV, Song Y, Wang J, Byon W, Boyd RA, Pursley JM, et al. Effect of famotidine on the pharmacokinetics of apixaban, an oral direct factor Xa inhibitor. Clin Pharmacol. 2013;5:59–66. doi: 10.2147/CPAA.S41999 23637566 PMC3637032

[29] Julkunen RJ. The absorption of warfarin from the rat stomach in situ. Med Biol. 1976;54(4):260–3. 8669

[30] Teichert M, van Noord C, Uitterlinden AG, Hofman A, Buhre PN, De Smet PA, et al. Proton pump inhibitors and the risk of overanticoagulation during acenocoumarol maintenance treatment. Br J Haematol. 2011;153(3):379–85. doi: 10.1111/j.1365-2141.2011.08633.x 21418179

[31] Henriksen DP, Stage TB, Hansen MR, Rasmussen L, Damkier P, Pottegard A. The potential drug-drug interaction between proton pump inhibitors and warfarin. Pharmacoepidemiol Drug Saf. 2015;24(12):1337–40. doi: 10.1002/pds.3881 26395871

[32] Wu AH. Drug metabolizing enzyme activities versus genetic variances for drug of clinical pharmacogenomic relevance. Clin Proteomics. 2011;8(1):12. doi: 10.1186/1559-0275-8-12 21906384 PMC3170273

[33] Wang KL, Lip GY, Lin SJ, Chiang CE. Non-Vitamin K Antagonist Oral Anticoagulants for Stroke Prevention in Asian Patients With Nonvalvular Atrial Fibrillation: Meta-Analysis. Stroke. 2015;46(9):2555–61. doi: 10.1161/STROKEAHA.115.009947 26304863 PMC4542566

[34] Abraham NS, Noseworthy PA, Yao X, Sangaralingham LR, Shah ND. Gastrointestinal Safety of Direct Oral Anticoagulants: A Large Population-Based Study. Gastroenterology. 2017;152(5):1014–22 e1. doi: 10.1053/j.gastro.2016.12.018 28043907

[35] Rutherford OW, Jonasson C, Ghanima W, Soderdahl F, Halvorsen S. Comparison of dabigatran, rivaroxaban, and apixaban for effectiveness and safety in atrial fibrillation: a nationwide cohort study. Eur Heart J Cardiovasc Pharmacother. 2020;6(2):75–85. doi: 10.1093/ehjcvp/pvz086 31942972 PMC7073510

[36] Mentias A, Heller E, Vaughan Sarrazin M. Comparative Effectiveness of Rivaroxaban, Apixaban, and Warfarin in Atrial Fibrillation Patients With Polypharmacy. Stroke. 2020;51(7):2076–86. doi: 10.1161/STROKEAHA.120.029541 32517580 PMC7388787

