# Supplementary material for: Risk of upper gastrointestinal bleeding in patients on oral anticoagulant and proton pump inhibitor co-therapy
Source: PLoS One. 2021 Jun 17;16(6):e0253310. doi: 10.1371/journal.pone.0253310 (PMC8211274; doi:10.1371/journal.pone.0253310)
Supplement: S1 File — S1 Fig, S1-S13 Tables. (PDF) [file pone.0253310.s001.pdf]

Risk of upper gastrointestinal bleeding

in patients on oral anticoagulant and proton pump inhibitor co-therapy

Hyun-Jung Lee[1], Hyung-Kwan Kim[1,*], Bong-Sung Kim[2], Kyung-Do Han[2], Jun-Bean Park[1], Heesun Lee[2], Seung-Pyo Lee[1], Yong-Jin Kim[1]

[1] Division of Cardiology, Department of Internal Medicine, Cardiovascular Center, Seoul National University Hospital, Korea

[2] Department of Statistics and Actuarial Science, Soongsil University, Seoul, Korea

Table of contents

1. **Supplementary Figure 1**
2. **Supplementary Tables 1-13**

Bleeding risk	OAC	N	Events	IR*	Adjusted HR† (95% CI)	p for interaction
**HAS-BLED = 0**	Warfarin	431	7	12.8	1 (ref.)	0.956
	Rivaroxaban	395	7	15.5	0.78 (0.26-2.32)	
	Dabigatran	112	0	0.0	-	
	Apixaban	99	0	0.0	-	
	Edoxaban	56	0	0.0	-	
**HAS-BLED = 1-2**	Warfarin	3086	82	16.4	1 (ref.)	
	Rivaroxaban	2830	63	17.3	0.70 (0.50-0.98)	
	Dabigatran	1055	19	18.0	0.65 (0.39-1.08)	
	Apixaban	1111	25	22.1	0.72 (0.45-1.14)	
	Edoxaban	677	10	13.9	0.49 (0.25-0.96)	
**HAS-BLED ≥ 3**	Warfarin	3478	117	19.1	1 (ref.)	
	Rivaroxaban	3243	104	20.7	0.97 (0.74-1.28)	
	Dabigatran	1134	29	20.5	0.90 (0.59-1.36)	
	Apixaban	1403	32	18.5	0.68 (0.46-1.02)	
	Edoxaban	741	17	20.0	0.75 (0.45-1.26)	

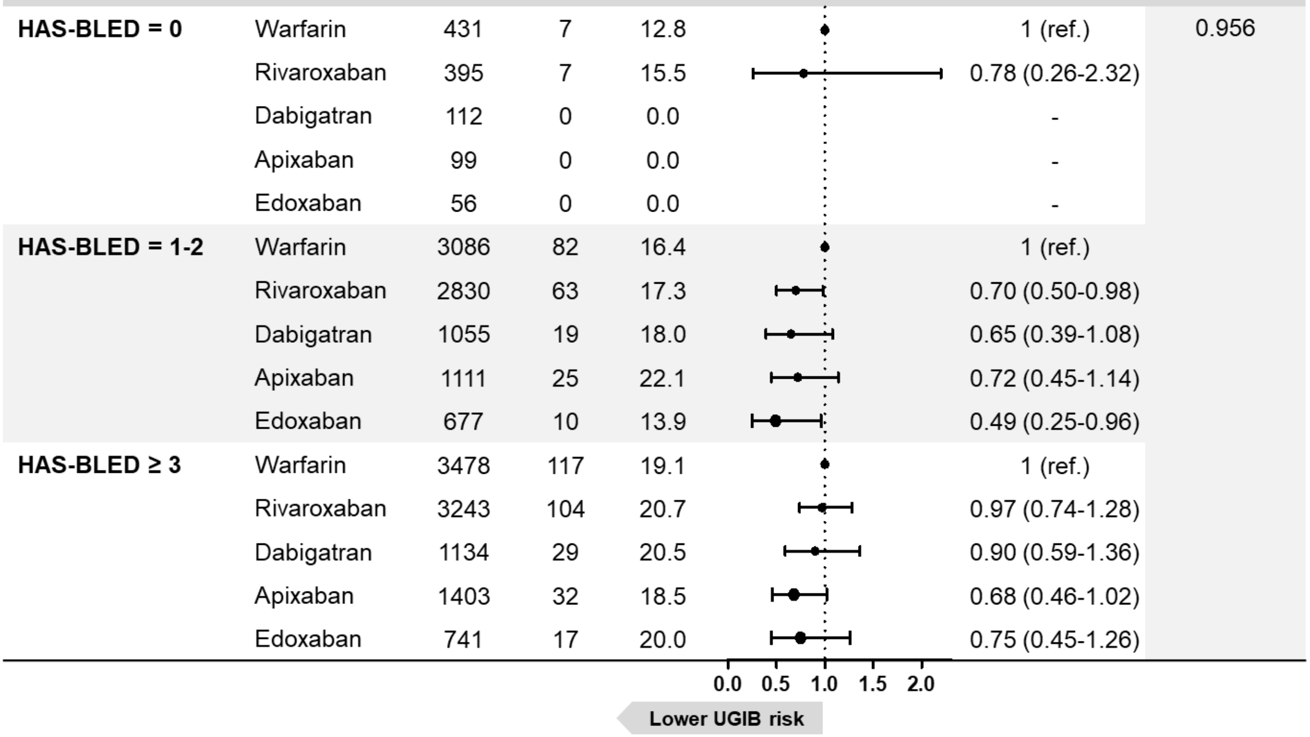

S1 Fig. Upper gastrointestinal bleeding risk of individual oral anticoagulants in subgroups according to bleeding risk

* IR: incidence rate per 1000 person-years

† Adjusted for age, sex, atrial fibrillation, venous thromboembolism, comorbidities (hypertension, diabetes mellitus, chronic kidney disease, ischemic heart disease, heart failure, peptic ulcer disease), and concomitant use of aspirin, P2Y12 inhibitor, and nonsteroidal anti-inflammatory drug.

UGIB, upper gastrointestinal bleeding

S1 Table. Definitions of comorbidities and outcomes

Variables	ICD-10 codes	Additional definitions
Covariates[*]		
Atrial fibrillation	I48	Admission ≥ 1 or outpatient clinic ≥ 2
Non-valvular atrial fibrillation	I48<br>Exclusion: I05.0, I05.2, I05.9 (rheumatic mitral stenosis); Z95.2-95.4 (presence of prosthetic heart valve)	Admission ≥ 1 or outpatient clinic ≥ 2
Venous thromboembolism	I26 (pulmonary thromboembolism), I80-82 (deep vein thrombosis)	Admission ≥ 1 or outpatient clinic ≥ 2
Hypertension	I10-I13, I15	Admission ≥ 1 or outpatient clinic ≥ 2<br>Minimum 1 prescription of anti-hypertensive drug (thiazide, loop diuretics, aldosterone antagonist, alpha-/beta-blocker, calcium-channel blocker, angiotensin-converting enzyme inhibitor, angiotensin II receptor blocker)

Diabetes mellitus	E11-E14	Admission ≥ 1 or outpatient clinic ≥ 2<br>Minimum 1 prescription of anti-diabetic drugs (sulfonylureas, metformin, meglitinides, thiazolidinediones, dipeptidyl peptidase-4 inhibitors, α-glucosidase inhibitors, SGLT2-inhibitor, GLP-1 agonist, or insulin)
Chronic kidney disease	N00-007, N11, I12, N18-19, Q61	Admission ≥ 1 or outpatient clinic ≥ 2
End-stage renal disease	N18.5, N18.9, N19, Z49, Z99.2	1) Dialysis ≥ 2<br>[Procedure codes: O7011-7020 (hemodialysis), O7071-O7075 (peritoneal dialysis)]<br>or 2) Registration for rare disease [V001 (hemodialysis), V003 (peritoneal dialysis)]
Kidney transplantation	Z94.0	Admission ≥ 1 or outpatient clinic ≥ 2
Liver disease	K70, K71, K72, K73, K74, K75.0-K75.8, K76.0-K76.7, B15-19	Admission ≥ 1 or outpatient clinic ≥ 2
Liver cirrhosis	K70.3, K74.3-74.6, K71.1, K76.1	Admission ≥ 1 or outpatient clinic ≥ 2
Ischemic heart disease	I20-25	Admission ≥ 1 or outpatient clinic ≥ 2
Heart failure	I50, I42.0, I11.0, I13.0, I13.2	Admission ≥ 1 or outpatient clinic ≥ 2

Cancer	C00-97	Admission ≥ 1 or outpatient clinic ≥ 2
GI cancer	C15-26	Admission ≥ 1 or outpatient clinic ≥ 2
Esophageal varix	I85	Admission ≥ 1 or outpatient clinic ≥ 2
Peptic ulcer	K25-27	Admission ≥ 1 or outpatient clinic ≥ 2
Gastritis and duodenitis	K29	Admission ≥ 1 or outpatient clinic ≥ 2
Gastroesophageal reflux disease	K21	Admission ≥ 1 or outpatient clinic ≥ 2
Endpoints		
Ischemic stroke	I63, I64	Admission ≥ 1 with claims for brain CT or MRI<br>(Procedure codes) HA441, HA451, HA461, HA851 (Brain CT);<br>HE101, HE201, HE301, HE401, HE501 (Brain MRI)
Intracranial hemorrhage	I60-62	Admission ≥ 1 with claims for brain CT or MRI
Admission for upper GI bleeding	K25.0, K25.2, K25.4, K25.6, K26.0, K26.2, K26.4, K26.6, K27.0, K27.2, K27.4, K27.6, K28.0, K28.2, K28.4, K28.6, K29.0, K92.0-92.2	Admission ≥ 1 with packed RBC transfusion ≥ 1<br>packed RBC codes: X2021, 2022, 2031, 2032, 2091, 2092, 2111, 2112, 2131, 2132

Admission for other GI bleeding	I85.0, K22.6, K55.2, K62.5, K63.3, K64.9	Admission ≥ 1 with packed RBC transfusion ≥ 1<br>packed RBC codes: X2021, 2022, 2031, 2032, 2091, 2092, 2111, 2112, 2131, 2132
Risk scores	*Components*	
HAS-BLED score[†]	Hypertension (1 point); Abnormal renal function (1 point; end-stage renal disease, chronic kidney disease, kidney transplantation); Abnormal liver function (1 point; liver cirrhosis, liver disease); Stroke (1 point; ischemic stroke); Bleeding (1 point; previous hospitalization for GI bleeding, peptic ulcer); Elderly (1 point; age > 65 years); Alcohol (1 point; > 8 times/week); Antiplatelet or NSAID use (1 point)	
CHA_2DS_2-VASc score	Congestive heart failure (1 point); Hypertension (1 point); Age (2 points if ≥ 75 years; 1 point if ≥ 65 years); Diabetes mellitus (1 point); Prior stroke or transient ischemic attack or systemic embolism (2 points); Vascular disease (1 point; myocardial infarction or peripheral artery disease); Female sex (1 point)	

*Covariates were defined by diagnoses during hospitalization or at outpatient clinic during the past 1 year.

[†] Labile prothrombin time was not included in the score due to lack of data.

CT, computed tomography; GI, gastrointestinal; ICD-10, International Classification of Diseases 10th revision from the World Health Organization; MRI, magnetic resonance imaging; RBC, red blood cell

S2 Table. Prescription of oral anticoagulants according to the index year of prescription

	Warfarin (n=6995)	Rivaroxaban (n=6468)	Dabigatran (n=2301)	Apixaban (n=2613)	Edoxaban (n=1474)
**Index year**					
2013	1622 (23.2)	193 (3.0)	22 (1.0)	0 (0)	0 (0)
2014	1854 (26.5)	584 (9.0)	8 (0.4)	5 (0.2)	0 (0)
2015	1530 (21.9)	1244 (19.2)	431 (18.7)	226 (8.7)	0 (0)
2016	1098 (15.7)	2176 (33.6)	904 (39.3)	934 (35.7)	344 (23.3)
2017	891 (12.7)	2271 (35.1)	936 (40.7)	1448 (55.4)	1130 (76.7)

S3 Table. Follow-up duration of each oral anticoagulant group

	Warfarin (n=6995)	Rivaroxaban (n=6468)	Dabigatran (n=2301)	Apixaban (n=2613)	Edoxaban (n=1474)
Follow-up durations throughout the whole study period					
**Upper GI bleeding**					
Median (IQR)	1.1 (0.3-2.7)	1.2 (0.3-2.2)	0.9 (0.2-1.8)	1.1 (0.3-1.8)	1.2 (0.4-1.7)
Mean ± SD	1.7 ± 1.7	1.4 ± 1.3	1.1 ± 1.0	1.1 ± 0.9	1.1 ± 0.7
**Death**					
Median (IQR)	1.1 (0.3-2.8)	1.2 (0.3-2.3)	0.9 (0.2-1.9)	1.1 (0.3-1.8)	1.2 (0.4-1.7)
Mean ± SD	1.7 ± 1.7	1.4 ± 1.3	1.1 ± 1.0	1.1 ± 0.9	1.1 ± 0.7
Follow-up durations censored at 2 years (sensitivity analysis I)					
**Upper GI bleeding**					
Median (IQR)	1.1 (0.3-2)	1.2 (0.3-2.0)	0.9 (0.2-1.8)	1.1 (0.3-1.8)	1.2 (0.4-1.7)
Mean ± SD	1.1 ± 0.8	1.1 ± 0.8	1.0 ± 0.8	1.0 ± 0.7	1.1 ± 0.7
**Death**					
Median (IQR)	1.1 (0.3-2.0)	1.2 (0.3-2.0)	0.9 (0.2-1.9)	1.1 (0.3-1.8)	1.2 (0.4-1.7)
Mean ± SD	1.1 ± 0.8	1.1 ± 0.8	1.0 ± 0.8	1.0 ± 0.7	1.1 ± 0.7

GI, gastrointestinal

S4 Table. Upper gastrointestinal bleeding risk according to individual NOACs

	N	Events	Crude HR (95% CI)	p-value	Overall p-value	Adjusted HR* (95% CI)	p-value	Overall p-value
Rivaroxaban	6468	174	1 (reference)		0.471	1 (reference)		0.332
Dabigatran	2301	48	0.89 (0.64-1.22)	0.456		0.91 (0.65-1.27)	0.581	
Apixaban	2613	57	0.90 (0.66-1.21)	0.482		0.80 (0.58-1.10)	0.168	
Edoxaban	1474	27	0.74 (0.49-1.11)	0.141		0.72 (0.47-1.10)	0.126	

* Adjusted for age, sex, atrial fibrillation, venous thromboembolism, comorbidities (hypertension, diabetes mellitus, chronic kidney disease, ischemic heart disease, heart failure, peptic ulcer disease), concomitant use of aspirin, $P2Y_{12}$ inhibitor, and nonsteroidal anti-inflammatory drug.

NOAC, non-vitamin K antagonist oral anticoagulant

S5 Table. Sensitivity analysis I (censoring at 2 years): Upper gastrointestinal bleeding risk according to oral anticoagulant treatment

	N	Events	IR*	Crude HR (95% CI)	p-value	Adjusted HR† (95% CI)	p-value
Warfarin	6995	174	22.7	1 (reference)	0.328	1 (reference)	0.011
NOACs	12856	287	21.1	0.91 (0.75-1.10)		0.78 (0.64-0.94)	
	N	Events	IR*	Crude HR (95% CI)	p-value	Adjusted HR† (95% CI)	p-value
Warfarin	6995	174	22.7	1 (reference)	0.360	1 (reference)	0.034
Rivaroxaban	6468	160	22.5	0.99 (0.80-1.23)		0.86 (0.69-1.07)	
Dabigatran	2301	47	21.1	0.88 (0.64-1.22)		0.79 (0.57-1.09)	
Apixaban	2613	55	20.6	0.87 (0.64-1.18)		0.69 (0.51-0.94)	
Edoxaban	1474	25	15.8	0.67 (0.44-1.02)		0.58 (0.38-0.88)	

* IR: incidence rate per 1000 person-years

† Adjusted for age, sex, atrial fibrillation, venous thromboembolism, comorbidities (hypertension, diabetes mellitus, chronic kidney disease, ischemic heart disease, heart failure, peptic ulcer disease), concomitant use of aspirin, $P2Y_{12}$ inhibitor, and nonsteroidal anti-inflammatory drug.

NOAC, non-vitamin K antagonist oral anticoagulant

S6 Table. Sensitivity analysis I (censoring at 2 years): Upper gastrointestinal bleeding risk according to individual NOACs

	N	Crude HR (95% CI)	p-value	Adjusted HR* (95% CI)	p-value
Rivaroxaban	6468	1 (reference)	0.336	1 (reference)	0.216
Dabigatran	2301	0.90 (0.65-1.24)		0.92 (0.66-1.29)	
Apixaban	2613	0.89 (0.65-1.20)		0.79 (0.57-1.09)	
Edoxaban	1474	0.68 (0.45-1.04)		0.67 (0.43-1.03)	

* Adjusted for age, sex, atrial fibrillation, venous thromboembolism, comorbidities (hypertension, diabetes mellitus, chronic kidney disease, ischemic heart disease, heart failure, peptic ulcer disease), concomitant use of aspirin, $P2Y_{12}$ inhibitor, and nonsteroidal anti-inflammatory drug.

NOAC, non-vitamin K antagonist oral anticoagulant

S7 Table. Sensitivity analysis II (accounting for the competing risk of death): Upper gastrointestinal bleeding risk according to oral anticoagulant treatment

	N	Crude HR (95% CI)	p-value	Adjusted HR* (95% CI)	p-value
Warfarin	6995	1 (reference)	0.172	1 (reference)	0.004
NOAC	12856	0.88 (0.74-1.06)		0.77 (0.64-0.92)	

	N	Crude HR (95% CI)	p-value	Adjusted HR* (95% CI)	p-value
Warfarin	6995	1 (reference)	0.377	1 (reference)	0.027
Rivaroxaban	6468	0.95 (0.77-1.16)		0.84 (0.68-1.04)	
Dabigatran	2301	0.85 (0.62-1.16)		0.76 (0.55-1.05)	
Apixaban	2613	0.84 (0.63-1.13)		0.68 (0.50-0.91)	
Edoxaban	1474	0.70 (0.47-1.05)		0.62 (0.41-0.93)	

* Adjusted for age, sex, atrial fibrillation, venous thromboembolism, comorbidities (hypertension, diabetes mellitus, chronic kidney disease, ischemic heart disease, heart failure, peptic ulcer disease), concomitant use of aspirin, $P2Y_{12}$ inhibitor, and nonsteroidal anti-inflammatory drug.

NOAC, non-vitamin K antagonist oral anticoagulant

S8 Table. Sensitivity analysis II (accounting for the competing risk of death): Upper gastrointestinal bleeding risk according to individual NOACs

	N	Crude HR (95% CI)	p-value	Adjusted HR* (95% CI)	p-value
Rivaroxaban	6468	1 (reference)	0.495	1 (reference)	0.334
Dabigatran	2301	0.90 (0.65-1.23)		0.91 (0.65-1.28)	
Apixaban	2613	0.90 (0.66-1.21)		0.80 (0.58-1.09)	
Edoxaban	1474	0.74 (0.49-1.12)		0.73 (0.48-1.11)	

* Adjusted for age, sex, atrial fibrillation, venous thromboembolism, comorbidities (hypertension, diabetes mellitus, chronic kidney disease, ischemic heart disease, heart failure, peptic ulcer disease), concomitant use of aspirin, $P2Y_{12}$ inhibitor, and nonsteroidal anti-inflammatory drug.

NOAC, non-vitamin K antagonist oral anticoagulant

S9 Table. Sensitivity analysis III (excluding patients on antiplatelets or NSAIDs): Upper gastrointestinal bleeding risk according to oral anticoagulant treatment

	N	Events	IR*	Crude HR (95% CI)	p-value	Adjusted HR† (95% CI)	p-value
Warfarin	3399	97	32.4	1 (reference)	0.175	1 (reference)	0.020
NOACs	6923	178	30.2	0.84 (0.66-1.08)		0.74 (0.58-0.95)	
	N	Events	IR*	Crude HR (95% CI)	p-value	Adjusted HR† (95% CI)	p-value
Warfarin	3300	97	30.4	1 (reference)	0.020	1 (reference)	0.034
Rivaroxaban	3272	104	36.8	1.06 (0.80-1.39)		0.88 (0.66-1.17)	
Dabigatran	1316	25	24.2	0.65 (0.42-1.02)		0.63 (0.41-0.99)	
Apixaban	1515	34	26.4	0.72 (0.49-1.07)		0.65 (0.43-0.96)	
Edoxaban	820	15	20.1	0.55 (0.32-0.94)		0.52 (0.30-0.90)	

* IR: incidence rate per 1000 person-years

† Adjusted for age, sex, atrial fibrillation, venous thromboembolism, comorbidities (hypertension, diabetes mellitus, chronic kidney disease, ischemic heart disease, heart failure, peptic ulcer disease).

NSAID, nonsteroidal anti-inflammatory drug; NOAC, non-vitamin K antagonist oral anticoagulant

S10 Table. Sensitivity analysis III (excluding patients on antiplatelets or NSAIDs): Upper gastrointestinal bleeding risk according to individual NOACs

	N	Crude HR (95% CI)	p-value	Adjusted HR* (95% CI)	p-value
Rivaroxaban	3272	1 (reference)	0.018	1 (reference)	0.268
Dabigatran	1316	0.62 (0.40-0.96)		0.76 (0.66-1.20)	
Apixaban	1515	0.69 (0.47-1.01)		0.77 (0.57-1.15)	
Edoxaban	820	0.52 (0.30-0.90)		0.63 (0.43-1.10)	

* Adjusted for age, sex, atrial fibrillation, venous thromboembolism, comorbidities (hypertension, diabetes mellitus, chronic kidney disease, ischemic heart disease, heart failure, peptic ulcer disease).

NSAID, nonsteroidal anti-inflammatory drug; NOAC, non-vitamin K antagonist oral anticoagulant

S11 Table. Risk of death according to individual NOACs

	N	Crude HR (95% CI)	p-value	Overall p-value	Adjusted HR* (95% CI)	p-value	Overall p-value
Rivaroxaban	6468	1 (ref.)		<0.001	1 (ref.)		0.025
Dabigatran	2301	0.72 (0.62-0.83)	<0.001		0.83 (0.71-0.97)	0.016	
Apixaban	2613	0.87 (0.76-0.99)	0.031		0.93 (0.81-1.07)	0.296	
Edoxaban	1474	0.70 (0.59-0.84)	<0.001		0.81 (0.67-0.96)	0.019	

* Adjusted for age, sex, atrial fibrillation, venous thromboembolism, comorbidities (hypertension, diabetes mellitus, chronic kidney disease, ischemic heart disease, heart failure, peptic ulcer disease), concomitant use of aspirin, $P2Y_{12}$ inhibitor, and nonsteroidal anti-inflammatory drug.

NOAC, non-vitamin K antagonist oral anticoagulant

S12 Table. Baseline characteristics of patients on anticoagulation for atrial fibrillation

	Warfarin (n=6995)	Rivaroxaban (n=6468)	Dabigatran (n=2301)	Apixaban (n=2613)	Edoxaban (n=1474)	p-value
**Age (year)**						
Mean±SD	69.8±12.3	73.8±10.8	72.51±10.37	74.73±10.25	73.74±10.3	<0.001
≥75	1919 (41.4)	1482 (53.9)	823 (47.2)	1229 (58.5)	625 (52.7)	
**Sex, male**	2342 (50.6)	1212 (44.0)	908 (52.1)	960 (45.7)	548 (46.2)	<0.001
**Income, low 20%**	1079 (23.3)	664 (24.1)	389 (22.3)	455 (21.7)	284 (24.0)	0.255
**Comorbidities**						
Hypertension	3158 (68.2)	2014 (73.2)	1259 (72.2)	1554 (73.9)	862 (72.7)	<0.001
Diabetes mellitus	1080 (23.3)	680 (24.7)	427 (24.5)	541 (25.7)	274 (23.1)	0.205
Chronic kidney disease	283 (6.1)	114 (4.1)	55 (3.2)	132 (6.3)	50 (4.2)	<0.001
Ischemic heart disease	1402 (30.3)	845 (30.7)	535 (30.7)	723 (34.4)	377 (31.8)	0.014
Heart failure	1252 (27.0)	824 (29.9)	492 (28.2)	705 (33.5)	385 (32.5)	<0.001
Peptic ulcer disease	1330 (28.7)	798 (29.0)	447 (25.6)	584 (27.8)	312 (26.3)	0.058
**Medications**						
Aspirin	1525 (32.9)	530 (19.3)	316 (18.1)	372 (17.7)	183 (15.4)	<0.001
P2Y12 inhibitor	930 (20.1)	408 (14.8)	254 (14.6)	318 (15.1)	163 (13.7)	<0.001
Number of antiplatelet agents						<0.001
0	2783 (60.1)	2010 (73.0)	1291 (74.0)	1562 (74.3)	903 (76.1)	
1	1243 (26.8)	546 (19.8)	336 (19.3)	390 (18.6)	220 (18.6)	
2	606 (13.1)	196 (7.1)	117 (6.7)	150 (7.1)	63 (5.3)	
NSAID	1257 (27.1)	784 (28.5)	435 (24.9)	508 (24.2)	318 (26.8)	0.006

Previous PPI use	2058 (44.4)	1520 (55.2)	850 (48.7)	1093 (52.0)	679 (57.3)	<0.001
**CHA_2DS_2-VASc score**	3.3±2.1	3.8±2.0	3.5±2.0	3.9±2.0	3.6±2.0	<0.001
**F/U duration (years)**	1.1 (0.3-2.6)	1.1 (0.3-1.9)	0.9 (0.2-1.8)	1.1 (0.3-1.7)	1.2 (0.4-1.7)	

NSAID, nonsteroidal anti-inflammatory drug; PPI, proton pump inhibitor

S13 Table. Stroke risk according to individual NOACs in patients with non-valvular atrial fibrillation

	N	Crude HR (95% CI)	p-value	Overall p-value	Adjusted HR† (95% CI)	p-value	Overall p-value
Rivaroxaban	2752	1 (ref.)		0.337	1 (ref.)		0.374
Dabigatran	1744	0.86 (0.62-1.19)	0.372		0.86 (0.62-1.20)	0.375	
Apixaban	2102	0.94 (0.70-1.27)	0.685		0.92 (0.68-1.23)	0.558	
Edoxaban	1186	0.70 (0.47-1.04)	0.077		0.71 (0.47-1.05)	0.087	

*IR: incidence rate per 1000 person-years

†Adjusted for age, sex, atrial fibrillation, venous thromboembolism, comorbidities (hypertension, diabetes mellitus, chronic kidney disease, ischemic heart disease, heart failure, peptic ulcer disease), concomitant use of aspirin, $P2Y_{12}$ inhibitor, nonsteroidal anti-inflammatory drug, and CHA_2DS_2-VASc score.
